# Lysine demethylase 4A: from chromatin to centrosomes

**DOI:** 10.1111/febs.70319

**Published:** 2025-10-30

**Authors:** Anushka Sharma, Ruth M. Kearney, Ciaran G. Morrison

**Affiliations:** ^1^ Centre for Chromosome Biology, School of Biological and Chemical Sciences University of Galway Ireland

**Keywords:** centriole, centrosome, chromatin, genome stability, lysine demethylation, mitosis

## Abstract

The lysine demethylase 4A (KDM4A) remodels chromatin by histone demethylation, regulating gene expression. In this issue, Chowdhury *et al.* report a previously undescribed localisation for KDM4A outside the nucleus, at the pericentriolar material around the centrosome. KDM4A interacts with centrosome proteins, and its loss or inhibition leads to centrosome amplification or fragmentation, which in turn perturbs mitosis and causes genome instability. These findings suggest a link between chromatin regulation and control of centrosome number, through the regulation of KDM4A‐dependent lysine methylation.

## Graphical abstract

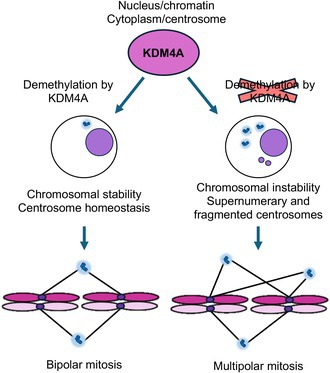

**Abbreviation**
PCM: pericentriolar material

## Introduction

Histone modifications are a major determinant of epigenetic gene regulation. Lysine‐specific demethylase 4A (KDM4A) is a histone demethylase belonging to the KDM4 family, members of which share conserved Jumonji N and C demethylase domains [1]. KDM4A removes di‐ and tri‐methyl groups from histone H3 (H3K9, H3K36) or histone H1 (H1.4 K26), thus regulating the transcription of a wide range of genes [1], as well as potentially impacting other chromatin transactions, such as DNA replication and repair. In this issue of the *FEBS Journal*, Chowdhury *et al*. uncover a previously undescribed centrosomal localisation for KDM4A and a role in the maintenance of centrosomal integrity, extending KDM4A's functions beyond its established nuclear role in gene regulation [2].

Centrosomes are the principal microtubule organising centres of animal cells, and form the poles of the mitotic spindle. Mitotic centrosomes comprise a pair of centrioles surrounded by the pericentriolar material (PCM). Successful cell division requires the formation of a bipolar spindle so that normal cells have two centrosomes to ensure accurate mitotic chromosome segregation. However, cancer cells frequently acquire supernumerary centrosomes, which may cause spindle multipolarity and other mitotic problems [3]. Thus, regulation of centrosome number and stability is important for minimising genome instability and avoiding cellular transformation.

## KDM4A is required for centrosomal integrity

In their study, Chowdhury *et al*. determined that KDM4A localises to the PCM and used co‐immunoprecipitation experiments to demonstrate that it can interact with centriolar proteins. Using CRISPR editing, siRNA and JIB‐04, a broad Jumonji histone demethylase inhibitor, they examined the consequences of KDM4A depletion and inhibition. Cells that lacked KDM4A showed centrosome amplification and fragmentation, accompanied by mitotic spindle defects and increased levels of genome instability, as demonstrated by the formation of micronuclei. Notably, the centrosome amplification was not accompanied by extended cell cycle arrest, suggesting a direct role for KDM4A in centrosome regulation. A rescue experiment demonstrated that wild‐type, but not catalytically inactive KDM4A could mitigate the observed genome instability, indicating the need for KDM4A's catalytic activity in regulating normal centrosome functioning. Notably, RNA‐sequencing analyses suggested that *KDM4A* deficiency did not differentially impact the expression of mitosis or centrosome‐related genes, suggesting that KDM4A's role in centrosome integrity is distinct from its functions in epigenetic regulation. These findings are interesting because they imply a role for KDM4A in centrosome regulation, potentially linking chromatin regulatory functions to centrosome numerical control.

## Centrosomal functions of KDM4A

Identification of the centrosomal targets of KDM4A demethylase activity is a key question that must be addressed to clarify the mechanism(s) through which it impacts centrosome integrity. Lysine methylation occurs on hundreds of nonhistone proteins throughout the nucleus and cytoplasm [4], so there are many potential mechanisms by which KDM4A may affect centrosomes.

**Fig. 1:**
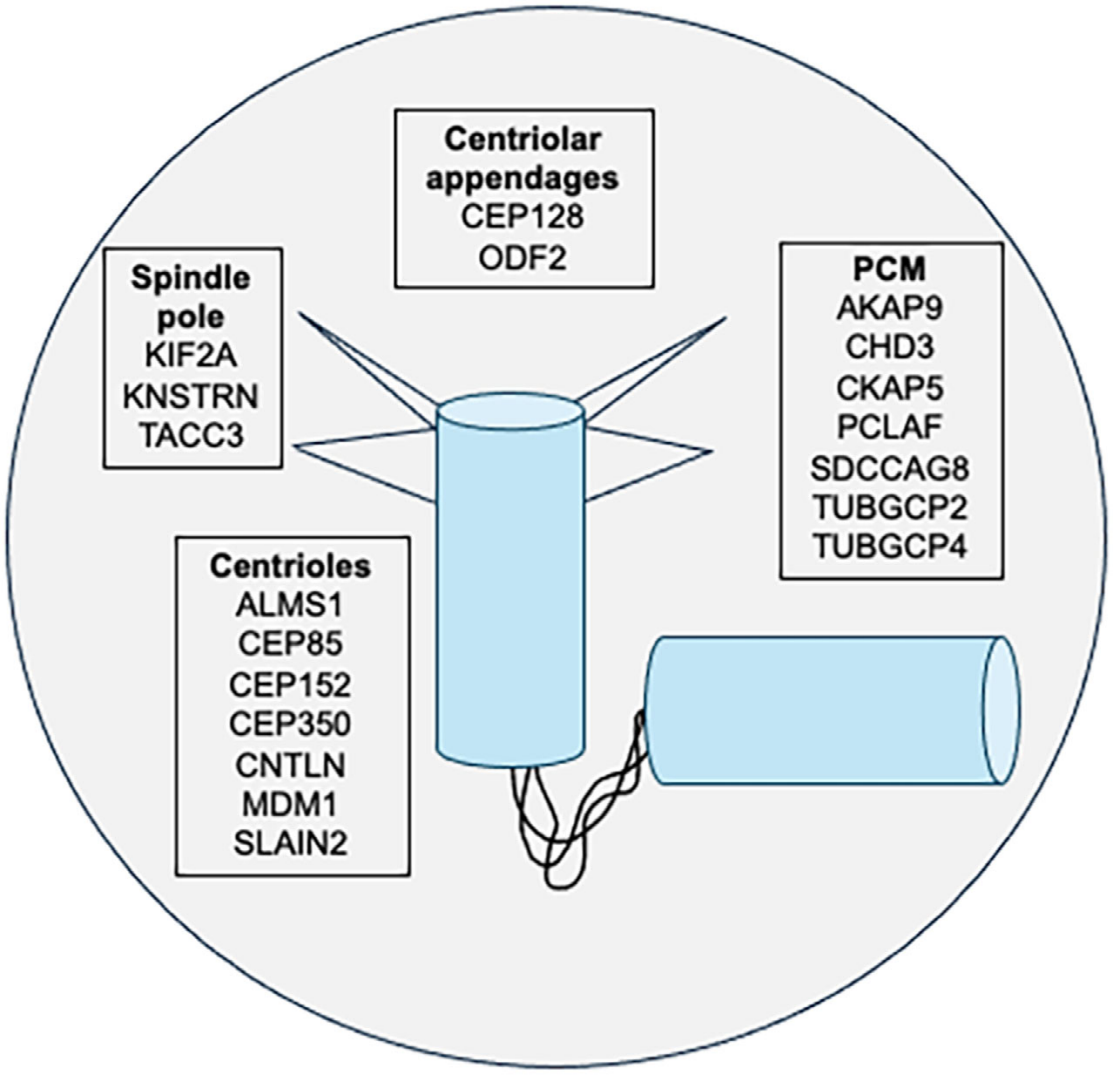
Centrosome localisation of potential KDM4A demethylation targets. Diagrammatic representation of the localisation of centrosome proteins (from the Human Protein Atlas), which are found on a list of experimentally validated lysine methylated human proteins (https://cplm.biocuckoo.cn/index.php). Note that the subcentrosomal positions indicated are not unique, as many of these localisations are dynamic and some have not been fully defined.

First, KDM4A may demethylate lysine residues on centrosomal target proteins. Protein–protein interaction analysis using STRING (https://string‐db.org/) identified KDM4A interactors at the centrosome. Additionally, yeast homologues of CHD3 and RUVBL2 were identified as potential interactors of KDM4A through two‐hybrid screening [5, 6]. CHD3 controls pericentrin positioning, while RUVBL2 regulates γ‐tubulin complex assembly [7, 8], either of which may contribute to the PCM organisation defects observed upon KDM4A depletion. Furthermore, CHD3 depletion produces spindle positioning defects. Considering potential centriolar targets for KDM4A regulation, affinity chromatography and interaction studies have detected interactions between the yeast KDM4A and the POC1B and KAT2A homologues [9, 10]. KAT2A has been shown to acetylate the centriolar kinase PLK4, regulating its activity and preventing centrosome overduplication [11], and POC1B is a structural protein essential for centriole integrity [12]. Interestingly, 23 of the 687 centrosomal proteins identified by the Human Protein Atlas (https://www.proteinatlas.org/) are also on a list of 1684 experimentally validated lysine methylated human proteins (https://cplm.biocuckoo.cn/index.php). As shown in Fig. 1, many of these centrosomal candidates for regulation by lysine methylation are localised to the PCM or subregions of the centrioles, providing support for the idea that KDM4A may demethylate its substrates within these regions of the centrosome. However, the large number of potential substrates also illustrates the difficulty of conclusively defining the mechanisms by which KDM4A affects centrosomes.

Centrosomal defects are seen to occur rapidly upon treatment with JIB‐04, suggesting that KDM4A inhibition causes centrosome fragmentation through a mechanism independent of epigenetic regulation of genes that encode centrosome proteins. However, since JIB‐04 does not specifically target KDM4A, and specific molecular inhibitors remain unavailable for the KDM4 family as yet, future studies may benefit from considering conditional approaches to examine KDM4A‐specific effects. Additionally, super‐resolution microscopy could reveal whether KDM4A loss affects specific centrosomal structural features, providing insights into how KDM4A regulates the centrosome.

An alternative possibility is that KDM4A acts on RNA‐binding proteins, rather than on centrosomal proteins themselves. KDM4A demethylation of RNA‐binding proteins may modulate their affinity for centrosomal mRNA targets or their capacity to recruit translation initiation complexes. Centrosomes contain specific mRNAs, such as *PCNT* and *CEP350* transcripts, that must be translated locally to build proper spindle structures [13]. KDM4A interacts with key components of the translation machinery, and its loss impacts protein synthesis [14], suggesting that KDM4A might demethylate RNA‐binding proteins to control local mRNA translation at centrosomes. For example, KDM4A directly interacts with RNA‐binding proteins hnRNPUL1 and FUS/TLS, both key regulators of RNA metabolism [15].

## Interplay between chromatin and centrosomal functions of KDM4A

An important theme arising from this study is the potential communication between the chromatin‐regulatory and centrosomal functions of the KDM4A demethylase—altered chromatin regulation may impact centrosomes, and vice versa. KDM4A overexpression in cancer is driving the development of therapeutic inhibitors that are designed to target its transcriptional functions. However, the current description of KDM4A's centrosomal role suggests that its inhibition may disrupt both gene expression and centrosome integrity. Cancer cells frequently harbour centrosome abnormalities and may alter KDM4A expression to compensate for this stress. KDM4A inhibition could exploit this dependency, selectively forcing cancer cells with centrosome defects into potentially lethal multipolar divisions, while normal cells with intact centrosome control mechanisms would remain unaffected [1].

In conclusion, KDM4A functions within multiple cellular compartments including the nucleus, cytoplasm and centrosome. Further work is required to define its nonhistone substrates at and around the centrosome, and to understand how their lysine methylation is regulated, particularly within the PCM. Such studies will elucidate how KDM4A's centrosomal activities connect with its transcriptional roles and may ultimately direct therapeutic strategies for cancers in which KDM4A is dysregulated.

## Conflict of interest

The authors declare no conflict of interest.

## Author contributions

AS, RMK and CGM wrote the paper.
